# The effect of habitually large protein intake on renal function of strength athletes: an update

**DOI:** 10.1186/1550-2783-8-S1-P33

**Published:** 2011-11-07

**Authors:** Lonnie M Lowery, Allison Daugherty, Brian Miller, Sarah Dye, Loren Liming

**Affiliations:** 1 Health, Exercise and Rehabilitation Sciences, Winona State University, 362 Maxwell Hall, Winona, MN 55987, USA; 2 Nutrition, Exercise and Wellness Associates, LLC, Cuyahoga Falls, OH 44223, USA; 3 Food Science and Human Nutrition, University of Maine, Orono, ME, USA; 4 The University of Akron Nutrition Center student volunteer, Akron, OH, USA

## Background

A quasi-experimental study was performed to evaluate the renal effects of large, chronic protein intakes among strength athletes. Population-specific data are still lacking regarding this cohort of athletes who commonly seek additional protein for performance and body composition purposes.

## Methods

In a design involving self-report, estimated and direct measures, urinary variables of renal function (extrapolated morning fasted- and actual 12-hour creatinine clearance) were assessed via a DCA 2000 urinalysis analyzer (Bayer healthcare, LLC, Elkhart, IN) in Caucasian male strength trainers (N=17) who do (PROT) and do not (CTRL) seek ample protein intake via foods and supplements. Use of fasted urinary Cr values in some creatinine clearance (CrCl) expressions allowed for comparisons beyond spot measures, without interference of daytime meals and mild activities. CrCl was derived as follows: U_Cr_ x U_vol_ / P_Cr_ x min. where U = urinary and P = plasma. Direct plasma variables (i.e. a standard “renal panel”) were also measured via commercial techniques (LabCare Plus, Barberton, OH) and compared. Body mass and composition were also assessed via balance scale and dual x-ray absorptiometry (DEXA). All participants abstained from exercise for three days prior to testing.

## Results

Over a reported 9.1+/-6.5 year period, chronic protein intakes (mean+/-SD: PROT 2.5 +/-0.83 g/kg, CTRL 1.27+/-0.33 g/kg), were greater in the protein seeking group (p < 0.05) as verified by diet logs. Concomitant with significantly greater 12-hour urine output (PROT 1811 +/-896 ml vs. CTRL 1162 +/-447 ml), no statistically significant effects were detected in creatinine clearance extrapolated from fasting urinary Cr values (PROT 255.0 +/-147.9 ml/min*1.73m^2^ vs. CTRL 196.5 +/-50.6 ml/min/1.73m^2^)or from actual 12-hour creatinine clearance (PROT 166.0 +/-59.1 ml/min*1.73m^2^ vs. CTRL 160.2 +/-38.5 ml/min/m^2^).Similarly, no differences were observed among serum variables including creatinine, BUN:Cr ratio, sodium, potassium, chloride, anion gap, calcium, albumin, or phosphorus. There was a trend toward higher BUN in PROT (8.8 +/-1.3 mg/dl vs. CTRL 8.4+/-1.8 mg/dl) but this disappeared when normalizing for serum creatinine. Groups did not differ in age but did differ in body mass (PROT 98.3 +/-16.8 kg vs. CTRL 83.3 +/-7.0 kg; p < 0.05) and fat free mass (PROT 79.0 +/-9.9 kg vs. CTRL 68.9 +/-6.7 kg; p < 0.05).

## Conclusion

It is concluded that, within the limitations of this research design, a multi-year intake of ample protein among male Caucasian strength athletes does not affect common markers of renal function. Future research should focus on long periods of high protein intake using true experimental designs, specific protein types, more sensitive renal function techniques such as inulin clearance, and any potential differences between protein foods versus supplements.

